# Investigation on the Efficiency of Chinese Herbal Injections combined with Concurrent Chemoradiotherapy for Treating Nasopharyngeal Carcinoma based on Multidimensional Bayesian Network Meta-analysis

**DOI:** 10.3389/fphar.2021.656724

**Published:** 2021-06-11

**Authors:** Zhishan Wu, Haojia Wang, Jiarui Wu, Siyu Guo, Wei Zhou, Chao Wu, Shan Lu, Miaomiao Wang, Xiaomeng Zhang, Jialin Li, Yingying Tan, Xiaotian Fan, Zhihong Huang

**Affiliations:** Department of Clinical Chinese Pharmacy, School of Chinese Materia Medica, Beijing University of Chinese Medicine, Beijing, China

**Keywords:** network meta-analysis, Bayesian model, nasopharyngeal carcinoma, Chinese herbal injections, multidimensional cluster

## Abstract

**Introduction:** Given the wide utilization of Chinese herbal injections in the treatment of nasopharyngeal carcinoma (NPC), this network meta-analysis (NMA) was devised to compare the clinical efficacy and safety of different Chinese herbal injections combined with concurrent chemoradiotherapy (CCRT) against NPC.

**Methods:** Randomized controlled trials (RCTs) were retrieved from seven electronic databases from the date of database establishment to October 5, 2020. Study selection and data extraction conformed to a priori criteria. Focusing on clinical effective rate, performance status, grade ≥3 oral mucositis, nausea and vomiting, leukopenia, and thrombopenia, this NMA was performed with Review Manager 5.3.5, Stata 13.1, WinBUGS 1.4.3, and R 4.0.3 software.

**Results:** Ten inventions from 37 RCTs involving 2,581 participants with NPC that evaluated the clinical effective rate, nausea and vomiting, leukopenia, thrombopenia, and grade ≥3 oral mucositis were included. Compared with CCRT alone, Elemene injection and Compound Kushen injection were associated with significantly improved clinical effective rates, and Elemene injection plus CCRT had the highest probability in terms of clinical effective rate (78.07%) compared with the other interventions. Shenqifuzheng injection, Xiaoaiping injection, and Shenmai injection ranked the best in terms of performance status (79.02%), nausea and vomiting (86.35%), and grade ≥3 oral mucositis (78.14%) when combined with CCRT. Kangai injection combined with CCRT ranked ahead of the other injections in terms of leukopenia (90.80%) and thrombopenia (91.04%), and had a better impact on improving performance status and reducing leukopenia, thrombopenia, grade ≥3 oral mucositis, and nausea and vomiting in the multidimensional cluster analysis.

**Conclusion:** Current clinical evidence indicates that Elemene injection combined with CCRT has the best clinical effective rate and that Kangai injection might have a comprehensively better impact on improving performance status and reducing adverse reactions against NPC. Additionally, due to the limitations of this NMA, more multicenter, high-quality, and head-to-head RCTs are needed to properly support our findings.

## Introduction

Nasopharyngeal carcinoma (NPC) is an uncommon malignant tumor compared with other cancers around the world. According to the report of the International Agency for Research on Cancer (Bray et al., 2018), there were 129,079 new cases of NPC and 72,987 deaths from NPC in 2018, accounting for 0.7 and 0.8% of the new cases and deaths of all cancers worldwide. However, NPC has geographical distribution characteristics and is endemic in north Africa, the Mediterranean basin, and southeast Asia, especially in southern China (Chang and Adami, 2006; Ferlay et al., 2015; Bray et al., 2018; Fu et al., 2018). NPC has aggressive locoregional spread along with a high rate of distant metastases among head and neck cancers (Wolden et al., 2006; Wu et al., 2016). Due to the anatomical limitations and high radiosensitivity of NPC, radiotherapy is the foundation of treatment. For locoregionally advanced NPC, guidelines of head and neck cancers (version 2.2020) from the National Comprehensive Cancer Network recommend a combination of concurrent chemoradiotherapy (CCRT) with either induction or adjuvant chemotherapy based on patient characteristics (Pfister et al., 2020). Although patients with NPC have significant survival benefits under current medical conditions, the adverse reactions (ARs) caused by radiotherapy and chemotherapy cannot be ignored (Xiao et al., 2011). Toxic effects such as leukopenia, nausea and vomiting, and other ARs reduce patients’ quality of life and may even lead to treatment incompletion (Frikha et al., 2018).

As a complementary and alternative medicine, Chinese medicine has gradually become accepted as an adjuvant treatment for cancers (Bao et al., 2014; Xiang et al., 2019). According to an article (Wu et al., 2015), the most common type of cancer treated by Chinese patent medicines in China from 2008 to 2010 was NPC, and Chinese herbal injections (CHIs) account for the largest proportion of Chinese patent medicines. However, a network meta-analysis (NMA) of different CHIs combined with CCRT for NPC has not been previously performed and it was still unclear which CHIs combined with CCRT were the most effective and tolerable against NPC. Thus, the present study used NMA to provide evidence-based hierarchies for this topic. In this NMA, we retrieved studies on 16 CHIs that were adopted in the treatment of NPC, namely, Aidi injection, Chansu injection, Compound Kushen injection, Delisheng injection, Elemene injection, Huachansu injection, Huangqi injection, Kangai injection, Kanglaite injection, Lentinan injection, Shenfu injection, Shengmai injection, Shenmai injection, Shenqifuzheng injection, Xiaoaiping injection, and Yadanziyouru injection, to determine their efficacy.

## Methods

This study was conducted in accordance with the Preferred Reporting Items for Systematic Reviews and Meta-Analyses (PRISMA) Extension Statement for Reporting of Systematic Reviews Incorporating Network Meta-analyses of Health Care Interventions (Hutton et al., 2015). A completed PRISMA checklist is included in Supplementary Material S1 (PRISMA Checklist).

### Eligibility Criteria and Exclusion Criteria

The eligibility criteria for this study were based on the PICOS principles given in the Cochrane Handbook, including patient, intervention, comparison, outcome, and study design.

Studies were considered eligible for inclusion if the following criteria were met. Study type: Randomized controlled trials (RCTs) of CHIs combined with CCRT for the treatment of NPC. The article describes that “random” can be included, and the language was unrestricted. Patient: Patients with a definite pathological diagnosis of NPC with no limitations on stage, sex, race, or nationality. Intervention and comparison: Interventions involving any one Chinese herbal injection combined with CCRT for the treatment of NPC. The control group included CCRT, regardless of induction, adjuvant chemotherapy, or another Chinese herbal injection. There were no limitations on the dosages or treatment courses. Outcome: The primary effectiveness outcome was the clinical effective rate. The secondary outcome was performance status, which was assessed by the Karnofsky Performance Status (KPS), and the ARs outcomes were grade ≥3 radiation-induced oral mucositis, nausea and vomiting, leukopenia, and thrombopenia. Clinical effective rate = (number of complete response patients + number of partial response patients)/total number of patients × 100%. After treatment, an increase in the KPS score by more than 10 points was considered effective. With regard to ARs, the incidence of ARs = number of patients with ARs/total number of patients × 100%.

The exclusion criteria were as follows: (1) patients had any other primary tumor; (2) the interventions included other Chinese medicine treatments, such as other Chinese patent medicine, Chinese herbal decoction, acupuncture, and massage; (3) the administration of CHIs was non-intravenous; (4) duplicate literature; (5) did not report relevant outcomes; and (5) the full text was unavailable.

### Search Strategy

Seven electronic databases, including PubMed, Cochrane Library, Embase, Chinese National Knowledge Infrastructure, Chinese Biomedical Literature Database, Chinese Scientific Journals Full-text Database, and Wanfang Database, were searched from inception to October 5, 2020 for articles on the treatment of NPC with CCRT and CHIs. To obtain the relevant literature, the search strategies were constructed for two domains: (1) nasopharyngeal cancer and (2) CHIs. Articles were retrieved by the combination of medical subject heading (MeSH) and free-text keywords. The following terms for NPC were used: “Nasopharyngeal Neoplasms” (MeSH), “Nasopharyngeal Carcinoma” (MeSH), “Nasopharynx Neoplasm”, “Cancer of Nasopharynx”, “Nasopharynx Cancer”, “Nasopharyngeal Cancer”, “Carcinoma, Nasopharyngeal”, “Nasopharyngeal Carcinomas”. The following terms for Compound Kushen injection were used: “Compound Kushen”, “Fufangkushen”, and “Compound matrine” (matrine is the main composition of Compound Kushen injection). The detailed retrieval strategies are provided in Supplementary Material S2. In addition, there were no restrictions on the blinding methods, publication year, or language. The references of the relevant systematic reviews and meta-analyses were also checked.

### Literature Selection and Data Extraction

NoteExpress software (Wuhan University Library, Wuhan, China) was used to manage the literature and delete duplicate studies. Two investigators independently perused the titles to remove apparently irrelevant studies as well as reviews and animal experimental reports. Furthermore, they read the abstracts and full texts of the remaining studies to screen for potential studies according to the inclusion criteria and extracted data from eligible RCTs. Any divergences were resolved through discussion or by the third reviewer in the implementation process.

The data of the eligible studies were extracted into a predesigned Microsoft Excel sheet. The main components of the extracted data were as follows: (1) first author and publication year; (2) baseline characteristics, i.e., sample size, sex, TNM stage, median age, average age or age range; (3) information on the intervention: dosage, course, and treatment cycle; and (4) outcomes and outcome measurement data of interest to the study.

### Risk of Bias Assessment

Two researchers assessed the risk of bias within individual studies independently by using the Cochrane Risk of Bias Tool recommended by the Cochrane Handbook 5.1 (Higgins et al., 2011). The items evaluated were as follows: (1) random sequence generation (selection bias); (2) allocation concealment (selection bias); (3) blinding of participants and personnel (performance bias); (4) blinding of outcome assessment (detection bias); (5) incomplete outcome data (attrition bias); (6) selective reporting (reporting bias); and (7) other bias. Three levels of bias were used to assess each of these items: “low risk,” “unclear risk,” and “high risk”. Discrepancies were resolved either by consensus or consultation with a third investigator.

### Statistical Analysis

STATA 13.1 software (Stata Corp, College Station, TX, United States), WinBUGS 1.4.3 software (Medical Research Council Biostatistics Unit, Cambridge, United Kingdom), and R 4.0.3 software (Mathsoft, Cambridge, United States) were used for statistical analysis. In this research, the outcomes were all dichotomous variables, and the odds ratio (OR) and its 95% confidence intervals (95% CI) were used to describe the effect. If the 95% CI did not contain one, the differences between the compared groups were statistically significant. The quality of the included RCTs was evaluated by Review Manager 5.3, and the NMA was carried out by WinBUGS software, while the Markov chain Monte Carlo method with a random-effects model was performed for Bayesian inference. The random-effects model for outcomes was chosen in the NMA. In WinBUGS software, the number of simulation iterations was 200,000, and the first 10,000 iterations were used for burn-in to eliminate the impact of the initial value (Crainiceanu and Goldsmith, 2010). Additionally, Stata version 13.1 software was adopted to analyze the results and draw the graphs of the NMA (Chaimani et al., 2013). The lines thickness corresponded to the number of trials used for the comparisons and the node sizes were weighted according to the total sample sizes of each treatment in the network graph. The results of WinBUGS software calculations were employed by Stata software to calculate the surface under the cumulative ranking curve (SUCRA). The “gemtc” package in R 4.0.3 software was used to analyze and visualize the NMA results of the clinical effective rate because the WinBUGS code could not analyze the rate when it was 100%. An intervention with a larger SUCRA value was considered to be the more effective treatment (Trinquart et al., 2016). Therefore, SUCRA was used to evaluate the ranking probabilities for each treatment. Publication bias was described *via* a comparison-adjusted funnel plot by Stata software (Trinquart et al., 2012). Symmetric points in the graph indicate that there is no obvious publication bias. Cluster analysis was also performed to comprehensively compare the effect of CHIs on two different outcomes, and the interventions located in the upper-right corner were superior to others (Veroniki et al., 2015).

### Multidimensional Cluster Analysis

Multidimensional cluster analysis based on the SUCRA values of any three outcomes of different CHIs was performed with the “scatterplot3d” package in R 4.0.3 software to comprehensively assess efficacy. The K-means method was adopted to cluster these interventions, and the number of clusters was modified according to the actual situation. The steps of clustering were as follows: (1) The included interventions were randomly divided into k initial categories, and the initial aggregation points were the average of the outcome of these k categories. (2) An intervention was classified into the closest aggregation point category and then the aggregation points of the category were updated to the average of the current outcome indicators. All interventions were recategorized and classified, and step (2) was repeated until all interventions were assigned. Finally, the ranking of the interventions for the three outcome indicators was visualized with a three-dimensional stereogram.

## Results

### Literature Retrieval and Screening Result

**FIGURE 1:**
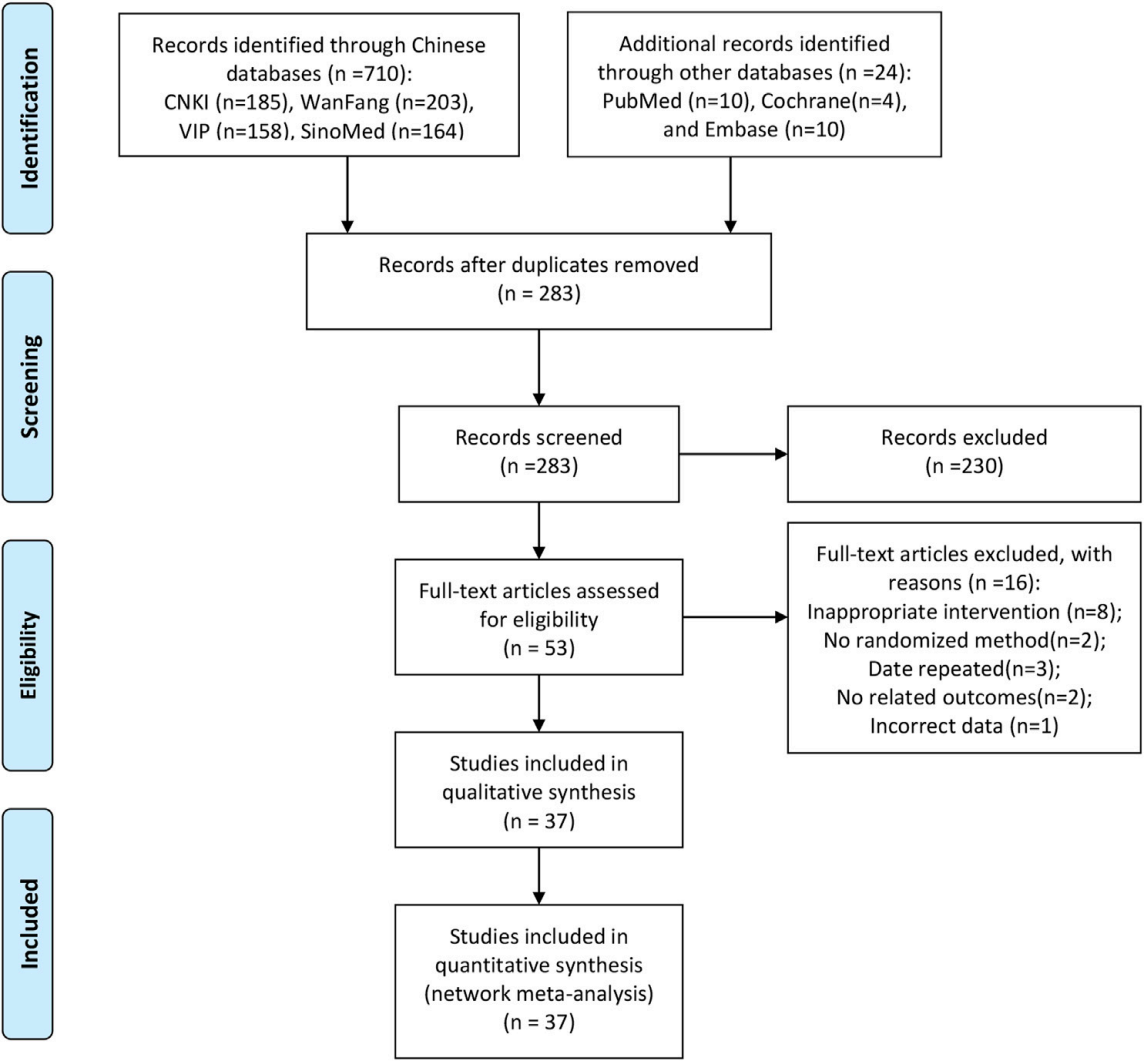
Flow chart of the search for eligible studies. Note: (*n*, number of articles. CNKI, China National Knowledge Infrastructure; WanFang, the WanFang Database; VIP, the Chinese Scientific Journals Full-Text Database; and SinoMed, the Chinese Biomedical Literature Database).

Initially, a total of 734 studies were retrieved using the search strategies. After removing duplicates and irrelevant articles, 283 studies remained, and through further inspection, a total of 37 RCTs involving nine CHIs met our selection criteria. The PRISMA flow diagram of study selection is shown in Figure 1. All of the studies were two-arm studies. The interventions of the 37 studies were CHIs plus CCRT, including nine kinds of CHIs, namely, Compound Kushen injection, Aidi injection, Shenqifuzheng injection, Kangai injection, Kanglaite injection, Shengmai injection, Elemene injection, Xiaoaiping injection, and Shenmai injection; the numbers of RCTs related to these medicines were 12, 6, 6, 2, 1, 2, 4, 3, and 1, respectively. Information about the included injections is shown in Supplementary Material S3. All studies were published in Chinese from 2004 to 2020.

### Characteristics of the Included Studies

**FIGURE 2:**
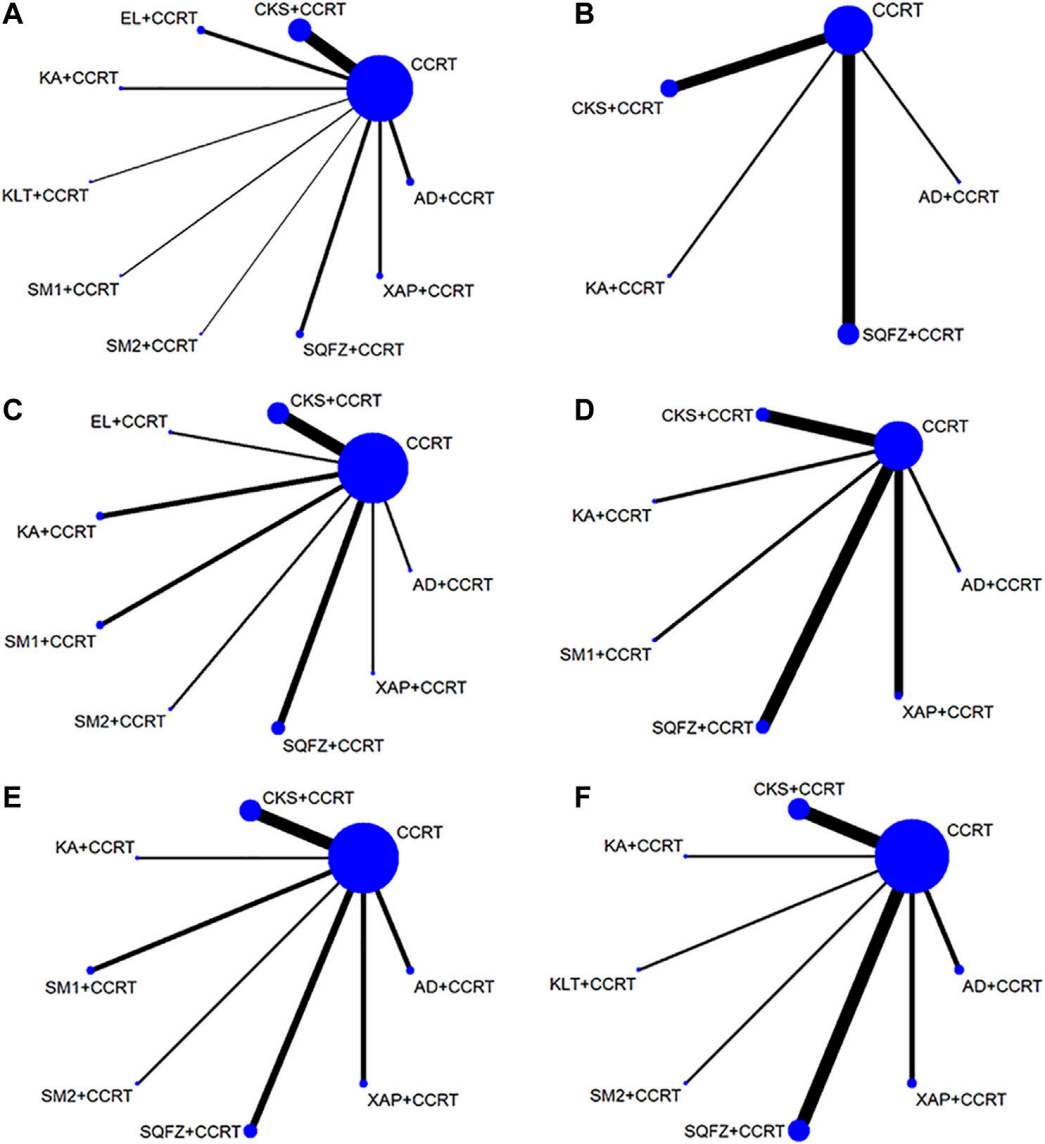
Network graph for different outcomes. **(A)** Clinical effective rate, **(B)** performance status, **(C)** leukopenia, **(D)** thrombopenia, **(E)** nausea and vomiting, and **(F)** grade ≥3 oral mucositis. Note: (CCRT, concurrent chemoradiotherapy; AD, Aidi injection; CKS, Compound Kushen injection; EL, Elemene injection; KA, Kangai injection; KLT, Kanglaite injection; SM1, Shengmai injection; SQFZ, Shenqqifuzheng injection; SM2, Shenmai injection; and XAP, Xiaoaiping injection).

The 37 RCTs with sample sizes varying from 30 to 142 included 2,851 patients and nine kinds of CHIs. Among them, 483 patients were treated with Compound Kushen injection + CCRT, 297 patients were treated with Aidi injection + CCRT, 168 patients were treated with Shenqifuzheng injection + CCRT, 64 patients were treated with Kangai injection + CCRT, 28 patients were treated with Kanglaite injection + CCRT, 78 patients were treated with Shengmai injection + CCRT, 148 patients were treated with Elemene injection + CCRT, 109 patients were treated with Xiaoaiping injection + CCRT, 81 patients were treated with Shenmai injection + CCRT, and 1,395 patients were treated with CCRT only. Except for one study (Hua et al., 2011) that did not report the sex composition, there were 1,811 male patients, accounting for 64.68% (1811/2800). Thirty-one (83.78%) studies reported the clinical effective rate, and 11 (29.73%), 17 (45.95%), 16 (43.24%), 16 (43.24%), and 11 (29.73%) RCTs reported the performance status, grade ≥3 radiation-induced oral mucositis, nausea and vomiting, leukopenia, and thrombopenia, respectively. The details of the study characteristics are provided in Table 1. The network graphs of the nine CHIs with different outcomes are depicted in Figure 2.

**TABLE 1:** The basic characteristics of the included studies.

References	TNM stages	KPS	Case, (A/B)	Sex, (M/F)	Average age (Year)	Intervention A	Intervention B	Course (d × c)	Outcomes
Xu et al. (2019)	III, IV	>70	30/30	36/24	A: 39–69 (54.18 ± 7.09)	IMRT/VMAT + DDP, DOC + CKS 20 ml, qd	IMRT/VMAT + DDP, DOC	(14 + 7) × 4	①
					B: 41–72 (54.69 ± 6.87)				
Jin et al. (2016a)	III, IV	>70	42/40	52/30	A: 19–64 (50.6, med)	IMRT + DDP + CKS 20 ml, qd	IMRT + DDP	(14 + 7) × 3	①
					B: 14–69 (47.8, med)				
Jin et al. (2016b)	III, IV	>70	42/40	52/30	A: 19–64 (50.6, med)	IMRT + DDP + CKS 20 ml, qd	IMRT + DDP	(14 + 7)× 3	⑥
					B: 14–69 (47.8, med)				
Liu et al. (2015a)	III, IV	≥70	30/30	35/25	A: 43, med B: 45, med	3D-CRT + NDP + CKS 20 ml, qd	3D-CRT + NDP	42	①②③⑤⑥
Wei (2015)	III, IV	NR	27/27	32/22	NR	2D-CRT + DDP + CKS 15 ml, qd	2D-CRT + DDP	(14 + 7) × 3	①
Wang and Zhou (2015)	III, IV	NR	45/45	49/41	A: 25–68 (50.5 ± 5.3)	2D-CRT + DDP + CKS 15 ml, qd	2D-CRT + DDP	49	①③④⑤
					B: 28–68 (50.5 ± 5.4)				
Song and Zhang (2014)	III, IV	NR	56/56	63/49	A: 30–60 (43, med)	IMRT + DDP, VIVA + CKS 15 ml, qd	IMRT + DDP + VIVA	(14 + 7) × 3	①③④⑤
					B: 31–62 (42, med)				
Fei et al. (2012)	III, IV	>70	60/60	75/45	A: 51.5 ± 11.1	2D-CRT + DDP, DOC + CKS 20 ml, qd	2D-CRT + DDP, DOC	42–49	①②⑤⑥
					B: 50.4 ± 9.0				
Xie et al. (2012)	III, IV	≥70	45/42	58/29	A: 18~58 (46.5)	2D-CRT + DDP + CKS 10 ml, qd	2D-CRT + DDP	49	①②③
					B: 21~60 (48.2)				
Wang et al. (2011)	NR	≥60	41/37	41/37	A: 34–76 (57, med)	2D-CRT + DDP, DOC/PTX + CKS 30 ml, qd	2D-CRT + DDP, DOC/PTX	21 × (2–3)	①②⑥
					B: 23–77 (53, med)				
Wei et al. (2011)	III	NR	30/30	40/20	NR	IMRT + DDP, DOC + CKS 20 ml, qd	IMRT + DDP, DOC	(15 + 5) × 3	①③④⑤
Cui et al. (2010)	III, IV	NR	35/35	56/14	A: 45, med B: 46, med	2D-CRT + DDP + CKS 15 ml, qd	2D-CRT + DDP	14	①⑥
Shi (2019)	III, IV	NR	30/30	37/23	A: 22–71 (49.5 ± 2.8)	2D-CRT + DDP + AD 50–100 ml, qd	2D-CRT + DDP	56	①
					B: 21–69 (48.7 ± 2.5)				
Wang et al. (2013)	III, IV	≥70	40/40	42/38	NR	2D-CRT + PLA + AD 60 ml, qd	2D-CRT + PLA	42 + (14 + 7) × 3	⑤⑥
Hu et al. (2013)	II, III, IV	≥80	60/56	89/27	NR	2D-CRT + DOC, 5-FU, DDP + AD 50–100 ml, qd	2D-CRT + DOC, 5-FU, DDP	56	①
Xiao et al. (2012)	II, III, IV	≥70	50/50	76/24	A: 21–73 (46.8)	IMRT + DDP, 5-FU + AD 50–80 ml, qd	IMRT + DDP, 5-FU	42	①
					B: 20–72 (47.3)				
Fu et al. (2010)	III, IV	≥70	37/36	54/19	A: 24–69 (47)	2D-CRT + DDP, 5-FU + AD 50 ml, qd	2D-CRT + DDP, 5-FU	42	③④⑤⑥
					B: 22–71 (46)				
Li et al. (2004)	III, IV	NR	80/76	118/38	A: 20–67 (47, med)	2D-CRT + DDP, 5-FU + AD 50 ml, qd	2D-CRT + DDP, 5-FU	42	①②
					B: 23–70 (49, med)				
Liu et al. (2015b)	III, IV	≥70	30/30	35/25	A: 27–69 (43, med)	3D-CRT + NDP + SQFZ 250 ml, qd	3D-CRT + NDP	42	①②③④⑤⑥
					B: 24–67 (45, med)				
Zhang C. Y. et al. (2014)	III, IV	≥70	32/30	47/15	A: 26–60 (45.6)	3D-CRT + DDP + SQFZ 250 ml, qd	3D-CRT + DDP	42	①②③④⑤⑥
					B: 23–60 (47.8)				
Yan et al. (2010)	NR	NR	16/14	18/12	NR	2D-CRT + DDP, 5-FU, CF + SQFZ 250 ml, qd	2D-CRT + DDP, 5-FU, CF	42–56	①②⑥
Xie and Wang (2010)	III, IV	≥60	30/30	37/23	A: 20–68 (51, med)	IMRT + DDP + SQFZ 250 ml, qd	IMRT + DDP	42	①②③④⑤⑥
					B: 9–66 (50, med)				
Zhang and Zhang (2009)	III, IV	≥70	30/28	44/14	A: 24–67 (49.5)	2D-CRT + DDP, 5-FU + SQFZ 250 ml, qd	2D-CRT + DDP, 5-FU	49	⑥
					B: 27–65 (50.0)				
Sun et al. (2005)	III, IV	≥80	30/30	44/16	NR	2D-CRT + DDP, 5-FU + SQFZ, 250 ml, qd	2D-CRT + DDP, 5-FU	(21 + 7) × 3	②
Liu et al. (2013)	NR	NR	35/35	39/31	A: 33–69 (49.2, med)	2D-CRT + DDP + KA, 40–60 ml, qd	2D-CRT + DDP	30>	①③
					B: 32–68 (47.8, med)				
Dai and Tang (2010)	III, IV	≥60	29/29	37/21	A: 29–71 (54)	2D-CRT + DDP + KA, 40 ml, qd	2D-CRT + DDP	49	①②③④⑤⑥
					B: 27–69 (63)				
Hua et al. (2011)	NR	≥80	28/23	NR	NR	2D-CRT + DDP + TXT KLT, 10 g/㎡	2D-CRT + DDP	(21 + 7) × 2	①⑥
Yang (2019)	III, IV	NR	52/52	59/45	A: 38–67 (48.67 ± 3.46)	IMRT + DDP + SM1, 50 ml, qd	IMRT + DDP	28	①③④⑤
					B: 35–69 (48.69 ± 3.43)				
Chen et al. (2017)	III, IV	≥80	26/25	30/21	A: 21–68 (46, med)	IMRT + DDP + SM1, 50 ml, qd	IMRT + DDP	28	①③⑤
					B: 18–70 (45, med)				
Zhang (2020)	Ⅰ, II	NR	41/41	44/38	A: 56–84 (69.56 ± 6.63)	IMRT + PTX, L-OPH + EL, 500 ml, qd	IMRT + DO	28	①③
					B: 54–86 (68.23 ± 7.01)				
Wu and Liu (2018)	Ⅰ, II	NR	40/40	49/31	A: 36–71 (57.7 ± 11.5)	2D-CRT + DDP, 5-FU + EL, 500 mg, qd	2D-CRT + DDP, 5-FU	28 × 2	①
					B: 36–73 (48.5 ± 2.3)				
Tian et al. (2017)	III, IV	≥70	24/24	29/19	A: 51.04 ± 8.97	IMRT + DDP + EL, 500 mg, qd	IMRT + DDP	(10 + 11) × 6	①
					B: 52.71 ± 7.80				
Zheng Q. H. et al. (2014)	Ⅰ, II	NR	43/43	52/34	A: 29–70 (47.9 ± 1.2)	2D-CRT + DDP, 5-FU + EL, 500 mg, qd	2D-CRT + PF	28 × 2	①
					B: 30–72 (48.5 ± 2.3)				
Zhou et al. (2015)	NR	NR	40/40	53/27	A: 50–75 (60.5 ± 2.5)	2D-CRT + PTX, NDP + XAP, 40 ml, qd	2D-CRT + PTX, NDP	(10 + 20) × 4	①④⑤⑥
					B: 51–78 (61.2 ± 2.1)				
Xiao et al. (2014)	NR	NR	30/30	43/17	NR	2D-CRT + PTX, NDP + XAP, 40 ml, qd	2D-CRT + PTX, NDP	(10 + 20) × 4	①③④⑤⑥
Zhou et al. (2009)	NR	≥70	39/30	48/21	A: 24–70 (47, med)	2D-CRT + DOC, DDP + XAP, 40 ml, qd	2D-CRT + TXT, DDP	NR	①
					B: 15–76 (45, med)				
Yao and Ye. (2009)	NR	NR	81/61	98/44	A: 37–65 (51)	2D-CRT + DDP + SM2, 50 ml, qd	IMRT + DDP	(21 + 7) × 2	②⑤⑥
					B: 39–70 (55)				

A, treatment group; B, control group; F, female; NR, not relate; M, male; Med, median; qd, once a day; 5-FU, 5-Fluorouracil; CF, calcium folinate; DDP, cisplatin; DOC, docetaxel; L-OPH, oxaliplatin; NDP, nedaplatin; PLB, platinum-based; PTX, paclitaxel; VIVA, vinorelbine; CCRT, concurrent chemoradiotherapy; AD, Aidi injection; CKS, Compound Kushen injection; EL, Elemene injection; KA, Kangai injection; KLT, Kanglaite injection; SM1, Shengmai injection; SQFZ, Shenqqifuzhen injection; SM2, Shenmai injection; and XAP, Xiaoaiping injection. ① clinical effective rate; ② performance status; ③ leukopenia; ④ thrombopenia; ⑤ nausea and vomiting; ⑥ grade ≥3 oral mucositis.

### Risk of Bias Assessment

**FIGURE 3:**
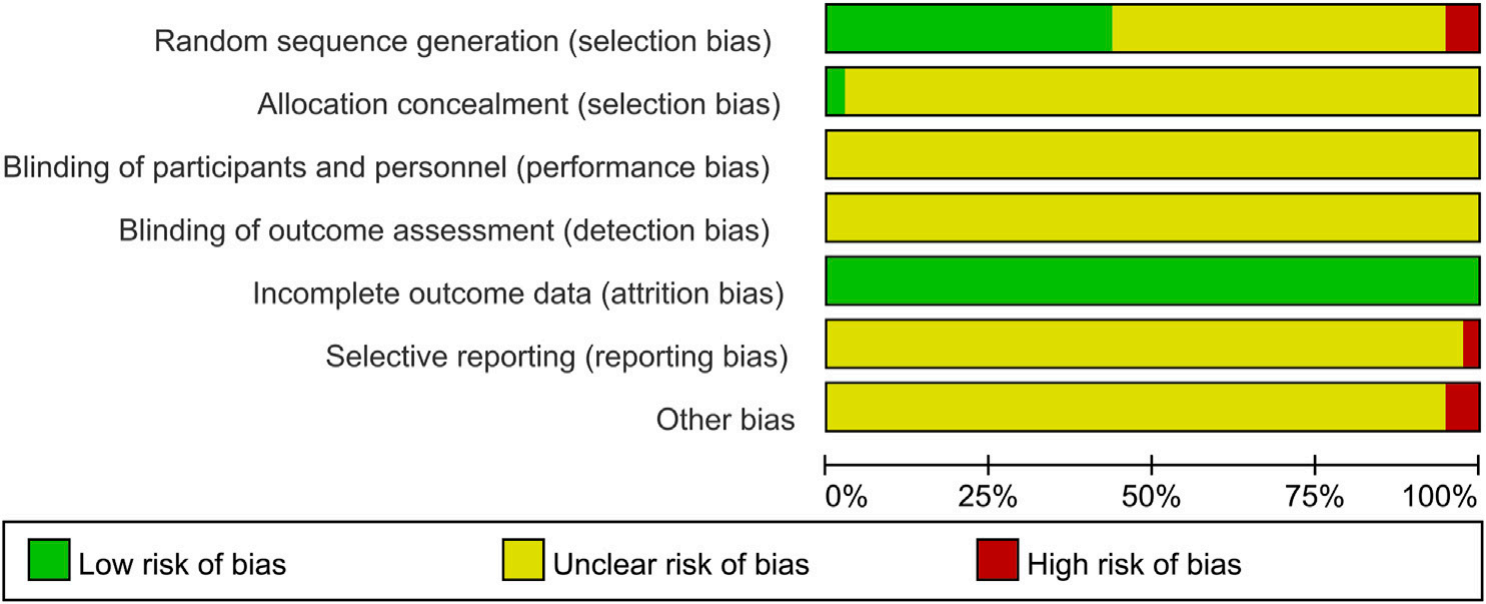
Assessment of risk bias.

In terms of random sequence generation, 16 of 37 studies used reasonable methods to generate the random sequence, including a random number table and envelope; thus, these studies were rated as low risk, and two trials (Wang et al., 2011; Liu et al., 2013) were regarded as high risk because the patients of the two groups were divided according to the admission time. None of the included studies mentioned information on allocation concealment and blinding. In terms of attrition bias, all studies had no incomplete data, so the evaluation was low risk. Regarding reporting bias, one study (Zhou et al., 2009) did not report the outcome data mentioned in the design plan and was considered to be high risk. Two studies (Yao and Ye, 2009; Xiao et al., 2014) did not describe the baseline conditions of the two groups as consistent, so the other biases were evaluated as high risk. In addition, the remaining studies were considered to have unclear risk due to insufficient information. The details of the risk of bias assessment for all included studies are shown in Figure 3.

### Results of the NMA

#### Clinical Effective Rate

A total of 31 studies with eight CHIs and nine interventions reported the clinical effective rate in the NMA. Compound Kushen injection (OR = 0.54; 95% CI, 0.29–0.96) and Elemene injection (OR = 0.32; 95% CI, 0.15–0.67) combined with CCRT were significantly more effective than CCRT alone. Combined with CCRT, Elemene injection might hold greater potential for increasing the clinical effective rate than Shenqifuzheng injection (OR = 3.71; 95% CI, 1.05–11.96). There were no statistically significant differences between the other interventions. The network graph is depicted in Figure 2, and the ORs with 95% CI are presented in Table 2 **.**

**TABLE 2:** Results of the NMA of clinical effective rate (upper-right quarter) and performance status (lower-left quarter).

CKS + CCRT	0.54 (0.29, 0.96)	1.10 (0.22, 6.89)	0.46 (0.15, 1.47)	1.02 (0.28, 3.73)	2.66 (0.18, 83.31)	1.06 (0.24, 5.03)	1.69 (0.64, 4.39)	6.7E−06 (3.3E−24, 29.5E+03)
0.56 (0.26, 1.21)	CCRT	0.50 (0.08, 2.19)	1.17 (0.43, 2.98)	0.53 (0.16, 1.59)	0.20 (0.01, 2.77)	0.50 (0.12, 1.97)	**0.32 (0.15, 0.67)**	8.4E+04 (1.8E−05, 1.6E+23)
1.38 (0.30, 6.66)	0.40 (0.10, 1.54)	AD + CCRT	0.43 (0.06, 2.58)	0.93 (0.12, 6.17)	2.40 (0.10, 93.53)	0.96 (0.11, 7.87)	1.57 (0.23, 8.52)	4.8E−06 (3.4E−24, 32.9E+03)
2.19 (0.77, 6.54)	**0.25 (0.12, 0.53)**	1.59 (0.34, 7.50)	SQFZ + CCRT	2.20 (0.49, 9.71)	5.75 (0.34, 194)	2.35 (0.43, 12.29)	**3.71 (1.05, 11.96)**	12.8E−06 (10.4E−24, 61.3E+03)
2.40 (0.41, 15.31)	0.23 (0.04, 1.16)	1.74 (0.21, 15.04)	1.09 (0.18, 6.78)	KA + CCRT	2.59 (0.16, 90.98)	1.05 (0.17, 6.22)	1.66 (0.42, 6.36)	5.5E−06 (4.2E−24, 29.9E+03)
–	–	–	–	–	KLT + CCRT	0.39 (0.01, 7.85)	0.64 (0.02, 9.81)	2.1E−06 (3.4E-24, 12.1E+03)
–	–	–	–	–	–	SM1+CCRT	1.56 (0.32, 7.65)	5.9E−06 (3.5E−24, 30.5E+03)
–	–	–	–	–	–	–	EL + CCRT	3.8E−06 (1.8E−24, 17.6E+03)
–	–	–	–	–	–	–	–	XAP + CCRT

The differences between the compared groups were deemed as significant when the 95% CI of the OR did not contain 1.00, which is marked as bold font. AD, Aidi injection; CCRT, concurrent chemoradiotherapy; CKS, Compound Kushen injection; EL, Elemene injection; KA, Kangai injection; KLT, Kanglaite injection; SM1, Shengmai injection; SQFZ, Shenqifuzheng injection; XAP, Xiaoaiping injection. Bold font described significant difference.

**FIGURE 4:**
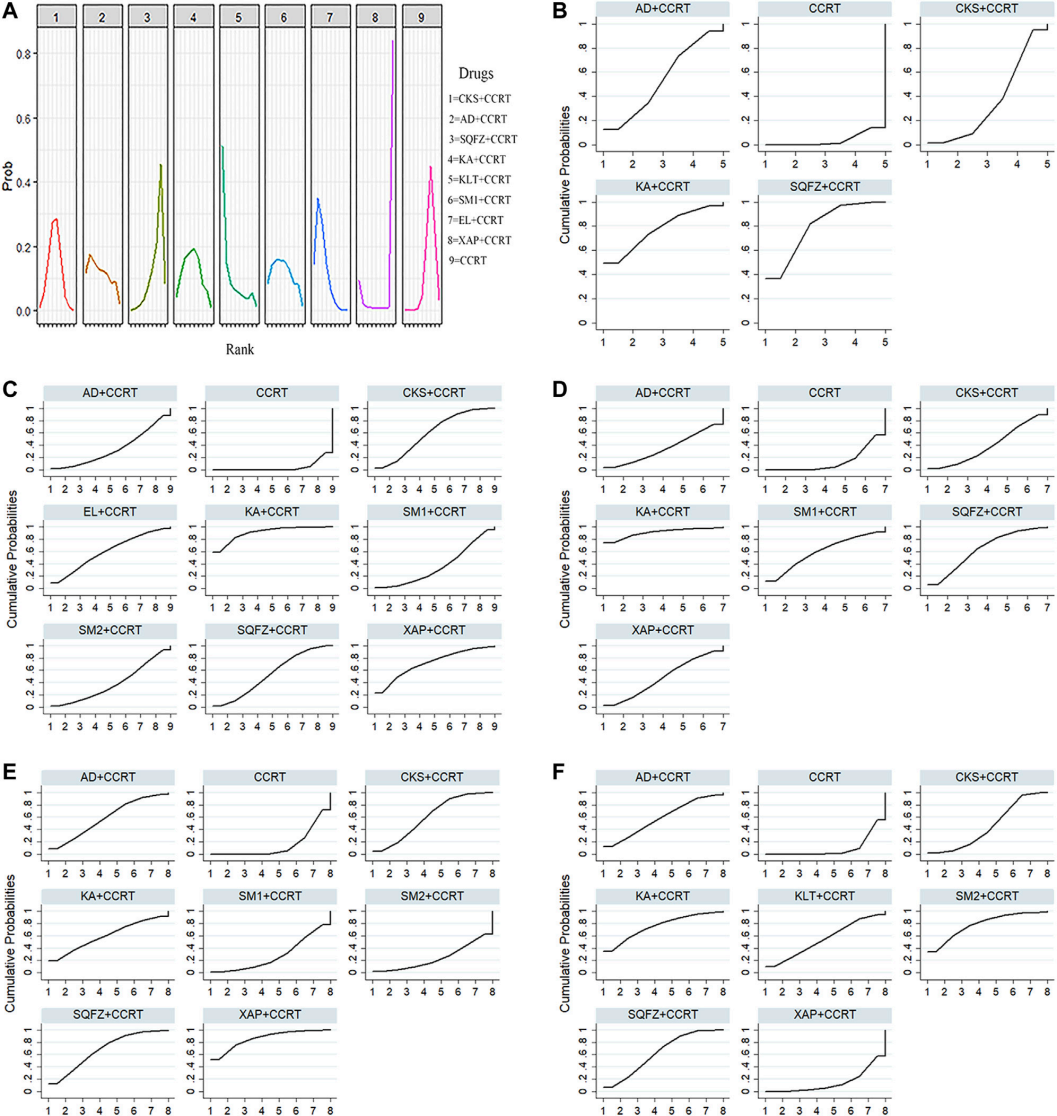
Rank probabilities and SUCRA for different outcomes. **(A)** The rank probability of clinical effective rate, **(B)** SUCRA of performance status, **(C)** SUCRA of leukopenia, **(D)** SUCRA of thrombopenia, **(E)** SUCRA of nausea and vomiting, and **(F)** SUCRA of grade ≥3 oral mucositis. Note: (AD, Aidi injection; CCRT, concurrent chemoradiotherapy; CKS, Compound Kushen injection; EL, Elemene injection; KA, Kangai injection; KLT, Kanglaite injection; SM1, Shengmai injection; SQFZ, Shenqifuzheng injection; SM2, Shenmai injection; and XAP, Xiaoaiping injection).

Based on the ranking result of the clinical effective rate, the relative ranking of interventions for improving the clinical effective rate was as follows: Elemene injection + CCRT (78.1%) > Kanglaite injection + CCRT (77.9%) > Aidi injection + CCRT (58.4%) > Shengmai injection + CCRT (57.4%) > Compound Kushen injection + CCRT (56.3%) > Kangai injection + CCRT (56.1%) > CCRT only (25.4%) > Shenqifuzheng injection + CCRT (23.5%) > Xiaoaiping injection + CCRT (16.9%). The results of the ranking probabilities are shown in Figure 4 and the ranking of SUCRA probabilities are shown in Table 3.

**TABLE 3:** Ranking probability of the various interventions among all interventions.

Intervention	Clinical effective rate		Performance status		Thrombopenia		Leukopenia		Nausea and vomiting		Grade ≥3 oral mucositis	
	SUCRA(%)	Rank	SUCRA(%)	Rank	SUCRA(%)	Rank	SUCRA(%)	Rank	SUCRA(%)	Rank	SUCRA(%)	Rank
CKS + CCRT	56.3	5	36.1	4	60.1	4	39.6	5	59.4	4	44.8	5-
AD + CCRT	**58.4**	3	**53.7**	3	34.3	8	34.9	6	58.4	5	57.6	4
SQFZ + CCRT	23.5	8	**79.0**	1	54.1	5	**63.5**	2	**67.4**	2	**63.3**	3
KA + CCRT	56.1	6	**77.3**	2	**90.8**	1	**91**	1	**60.9**	3	**77.3**	2
KLT + CCRT	**77.9**	2	–	–	–	–	–	–	–	–	55.2	–
SM1+CCRT	57.4	4-	–	–	36.4	7	**59.8**	3	27.9	–	–	–
EL + CCRT	**78.1**	1	–	–	**60.2**	3	–		–	–	–	–
XAP + CCRT	16.9	9	–	–	**71.5**	2	47.8	4	**86.4**	1	14.3	6
SM2+CCRT	–	–	–	–	38.5	6	–	–	24.7	6	**78.1**	1
CCRT	25.4	7	3.9	5	4.2	9	13.4	7	14.9	7	9.4	7

The surface under the cumulative ranking curve (SUCRA) was used to estimate the ranking probabilities for different CHIs. The values in bold font have higher SUCRA values for different outcomes. AD, Aidi injection; CCRT, concurrent chemoradiotherapy; CKS, Compound Kushen injection; EL, Elemene injection; KA, Kangai injection; KLT, Kanglaite injection; SM1, Shengmai injection; SM2, Shenmai injection; SQFZ, Shenqifuzheng injection; XAP, Xiaoaiping injection. Bold font described significant difference.

#### Performance Status

A total of 11 RCTs reported the improvement rate of the KPS score, involving four traditional CHIs and five interventions. The results of the NMA revealed that Shenqifuzheng injection combined with CCRT (OR = 0.25; 95% CI, 0.12–0.53) showed significant benefits in improving the KPS score. There were no statistically significant differences between the other interventions. The network graph is depicted in Figure 2, and the ORs with 95% CI are presented in Table 2.

Based on the ranking result of improving performance status, the relative ranking of interventions for improving the performance status was as follows: Shenqifuzheng injection + CCRT (79.0%) > Kangai injection + CCRT (77.3%) > Aidi injection + CCRT (53.7%) > Compound Kushen injection + CCRT (36.1%) > CCRT only (3.9%). The results of the ranking probabilities based on SUCRA are shown in Figure 4 and Table 3.

#### ARs

##### Leukopenia

A total of 16 studies with eight CHIs and nine interventions reported leukopenia in the NMA. Compound Kushen injection (OR = 3.71; 95% CI, 1.76–8.66), Shenqifuzheng injection (OR = 3.29; 95% CI, 1.37–8.08), Kangai injection (OR = 9.24; 95% CI, 2.99–33.68), and Xiaoaiping injection (OR = 5.37; 95% CI, 1.08–29.71) combined with CCRT were significantly more effective than CCRT alone. There were no statistically significant differences between the other interventions. The network graph is depicted in Figure 2 **,** and the ORs with 95% CI are presented in Table 4.

**TABLE 4:** Results of the NMA of thrombopenia (upper-right quarter) and leukopenia (lower-left quarter).

CKS + CCRT	1.68 (0.49, 7.35)	1.13 (0.10, 17.19)	0.56 (0.09, 4.14)	0.15 (0.01, 2.47)	0.57 (0.04, 8.94)	–	0.81 (0.12, 7.77)	–
**3.71 (1.76, 8.66)**	CCRT	1.48 (0.16, 13.31)	3.01 (0.81, 11.75)	**11.12 (1.10, 120)**	2.95 (0.31, 28.88)	–	2.08 (0.39, 10.02)	–
1.78 (0.36, 9.84)	2.08 (0.49, 8.98)	AD + CCRT	0.49 (0.04, 6.31)	0.13 (0.01, 3.14)	0.50 (0.02, 11.48)	–	0.71 (0.05, 11.32)	–
1.13 (0.36, 3.85)	**3.29 (1.37, 8.08)**	0.63 (0.12, 3.45)	SQFZ + CCRT	0.27 (0.02, 3.97)	1.02 (0.07, 14.10)	–	1.45 (0.19, 12.32)	–
0.40 (0.09, 1.68)	**9.24 (2.99, 33.68)**	0.22 (0.03, 1.41)	0.36 (0.07, 1.51)	KA + CCRT	3.76 (0.15, 99.55)	–	5.36 (0.34, 98.21)	–
1.64 (0.42, 6.26)	2.24 (0.79, 7.31)	0.93 (0.14, 5.34)	1.47 (0.33, 5.66)	4.12 (0.8, 21.25)	SM1+CCRT	–	1.43 (0.09, 24.52)	–
0.96 (0.19, 5.34)	3.86 (0.91, 17.23)	0.54 (0.07, 4.18)	0.85 (0.15, 4.75)	2.41 (0.38, 16.6)	0.58 (0.10, 3.9)	EL + CCRT	–	–
0.69 (0.11, 4.32)	**5.37 (1.08, 29.71)**	0.38 (0.04, 3.44)	0.62 (0.09, 3.81)	1.73 (0.22, 13.72)	0.42 (0.06, 3.14)	0.71 (0.08, 6.47)	XAP + CCRT	–
1.59 (0.34, 8.23)	2.33 (0.59, 9.54)	0.89 (0.12, 6.54)	1.42 (0.27, 7.36)	3.99 (0.66, 26.03)	0.96 (0.17, 6.10)	1.66 (0.22, 12.6)	2.31 (0.27, 20.53)	SM2+CCRT

The differences between the compared groups were deemed as significant when the 95% CI of the OR did not contain 1.00, which is marked as bold font. AD, Aidi injection; CCRT, concurrent chemoradiotherapy; CKS, Compound Kushen injection; EL, Elemene injection; KA, Kangai injection; SM1, Shengmai injection; SM2, Shenmai injection; SQFZ, Shenqifuzheng injection; XAP, Xiaoaiping injection. Bold font described significant difference.

Based on the ranking result of leukopenia, the relative ranking of interventions was as follows: Kangai injection + CCRT (90.8%) > Xiaoaiping injection + CCRT (71.5%) > Elemene injection + CCRT (60.2%) > Compound Kushen injection + CCRT (60.1%) > Shenqifuzheng injection + CCRT (54.1%) > Shenmai injection + CCRT (38.5%) > Shengmai injection + CCRT (36.4%) > Aidi injection + CCRT (34.3%) > CCRT only (4.2%). The results of the ranking probabilities based on SUCRA are shown in Figure 4 and Table 3.

##### Thrombopenia

A total of 11 studies with six CHIs and seven interventions reported thrombopenia in the NMA. Kangai injection (OR = 11.12; 95% CI, 1.10–120) combined with CCRT was significantly more effective than CCRT alone. There were no statistically significant differences between the other interventions. The network graph is depicted in Figure 2, and the ORs with 95% CI are presented in Table 4.

Based on the ranking results of thrombopenia, the relative ranking of interventions was as follows: Kangai injection + CCRT (91.0%) > Shenqifuzheng injection + CCRT (63.5%) > Shengmai injection + CCRT (59.8%) > Xiaoaiping injection + CCRT (47.8%) > Compound Kushen injection + CCRT (39.6%) > Aidi injection + CCRT (34.9%) > CCRT only (13.4%). The results of the ranking probabilities based on SUCRA are shown in Figure 4 and Table 3.

##### Nausea and Vomiting

A total of 16 studies with seven CHIs and eight interventions reported nausea and vomiting in the NMA. Compound Kushen injection (OR = 2.51; 95% CI, 1.13–5.80), Shenqifuzheng injection (OR = 2.99; 95% CI, 1.05–8.89), and Xiaoaiping injection (OR = 5.13; 95% CI, 1.45–22.83) combined with CCRT were significantly more effective than CCRT alone. There were no statistically significant differences between the other interventions. The network graph is depicted in Figure 2, and the ORs with 95% CI are presented in Table 5 **.**

**TABLE 5:** Results of the NMA of nausea and vomiting (upper-right quarter) and grade ≥3 oral mucositis (lower-left quarter).

CKS + CCRT	**2.51 (1.13, 5.8)**	1.03 (0.22, 4.63)	0.84 (0.22, 3.26)	0.94 (0.11, 7.76)	–	2.01 (0.45, 9.40)	0.49 (0.09, 2.19)	2.33 (0.37, 15.57)
**2.73 (1.28, 10.15)**	CCRT	2.44 (0.70, 9.36)	**2.99 (1.05, 8.89)**	2.67 (0.39, 19.26)	–	1.25 (0.35, 4.50)	**5.13 (1.45, 22.83)**	1.08 (0.20, 5.78)
0.69 (0.09, 8.48)	3.94 (0.55, 30.25)	AD + CCRT	0.82 (0.16, 4.52)	0.92 (0.09, 9.38)	–	1.95 (0.33, 12.55)	0.48 (0.07, 2.92)	2.27 (0.28, 19.65)
0.65 (0.14, 2.83)	**4.27 (1.66, 17.78)**	0.93 (0.07, 7.72)	SQFZ + CCRT	1.12 (0.12, 10.25)	–	2.40 (0.46, 12.86)	0.59 (0.09, 3.00)	2.77 (0.39, 20.37)
0.39 (0.04, 5.16)	6.98 (0.85, 67.69)	0.56 (0.03, 10.23)	0.61 (0.06, 8.86)	KA + CCRT	–	2.15 (0.21, 22.30)	0.52 (0.04, 5.18)	2.49 (0.20, 32.56)
0.78 (0.10, 9.58)	3.57 (0.48, 27.45)	1.11 (0.07, 19.11)	1.19 (0.14, 15.94)	1.96 (0.11, 41.49)	KLT + CCRT	–	–	–
–	–	–	–	–	–	SM1+CCRT	0.24 (0.03, 1.42)	1.16 (0.14, 9.50)
2.58 (0.50, 18.09)	1.07 (0.27, 5.15)	3.76 (0.28, 40.25)	3.98 (0.71, 29.97)	6.56 (0.44, 90.59)	3.35 (0.24, 37.54)	–	XAP + CCRT	4.73 (0.62, 46.71)
0.38 (0.06, 4.26)	**7.2 (1.10, 48.54)**	0.55 (0.04, 8.31)	0.58 (0.08, 7.12)	0.97 (0.06, 18.09)	0.49 (0.03, 7.91)	–	0.15 (0.01, 1.81)	SM2+CCRT

The differences between the compared groups were deemed as significant when the 95% CI of the OR did not contain 1.00, which is marked as bold font. AD, Aidi injection; CCRT, concurrent chemoradiotherapy; CKS, Compound Kushen injection; KA, Kangai injection; KLT, Kanglaite injection; SM1, Shengmai injection; SM2, Shenmai injection; SQFZ, Shenqifuzheng injection; XAP, Xiaoaiping injection. Bold font described significant difference.

Based on the ranking results of nausea and vomiting, the relative ranking of interventions was as follows: Xiaoaiping injection + CCRT (86.4%) > Shenqifuzheng injection + CCRT (67.4%) > Kangai injection + CCRT (60.9%) > Compound Kushen injection + CCRT (59.4%) > Aidi injection + CCRT (58.4%) > Shengmai injection + CCRT (27.9%) > Shenmai injection + CCRT (24.7%) > CCRT only (14.9%). The results of the ranking probabilities based on SUCRA are shown in Figure 4 and Table 3.

##### Grade ≥3 Oral Mucositis

A total of 17 studies with seven CHIs and eight interventions reported grade ≥3 oral mucositis in the NMA. Compound Kushen injection (OR = 2.73; 95% CI, 1.28–10.15), Shenqifuzheng injection (OR = 4.27; 95% CI, 1.66–17.78), and Shenmai injection (OR = 7.20; 95% CI, 1.10–48.54) combined with CCRT were significantly more effective than CCRT alone. There were no statistically significant differences between the other interventions. The network graph is depicted in Figure 2, and the ORs with 95% CI are presented in Table 5.

Based on the ranking result of grade ≥3 oral mucositis, the relative ranking of interventions was as follows: Shenmai injection + CCRT (78.1%) > Kangai injection + CCRT (77.3%) > Shenqifuzheng injection + CCRT (63.3%) > Aidi injection + CCRT (57.6%) > Kanglaite injection + CCRT (55.2%) > Compound Kushen injection + CCRT (44.8%) > Xiaoaiping injection + CCRT (14.3%) > CCRT only (9.4%). The results of the ranking probabilities based on SUCRA are shown in Figure 4 and Table 3.

### Cluster Analysis

**FIGURE 5:**
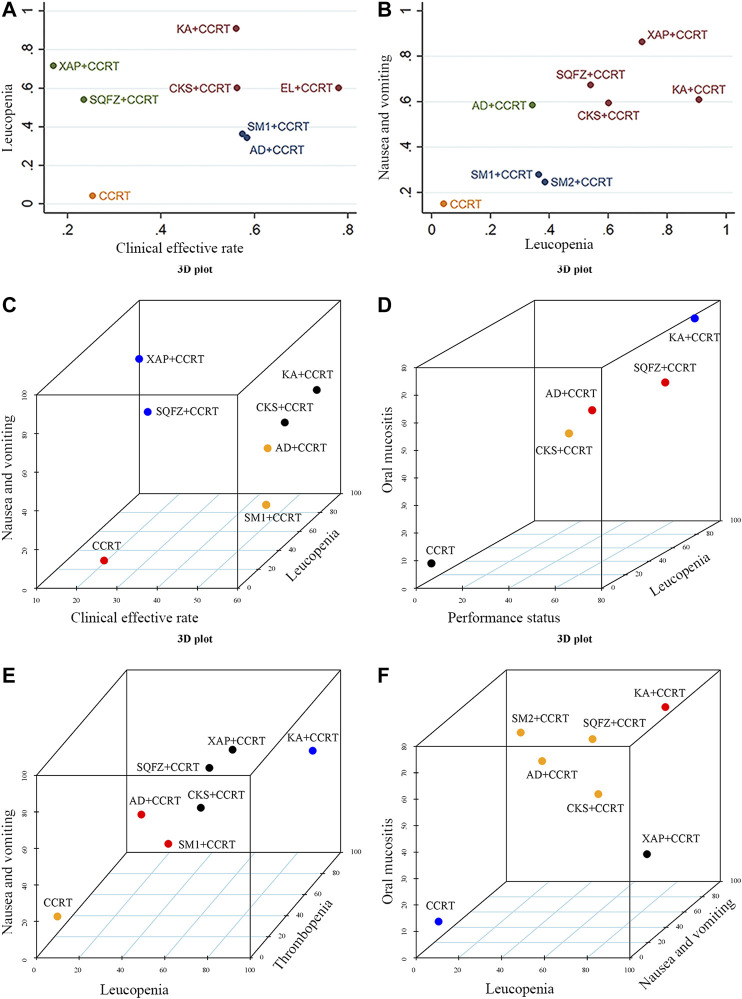
Cluster analysis plots for six outcomes. **(A)** Clinical effective rate (*x*-axis) and leukopenia (*y*-axis), **(B)** leukopenia and nausea and vomiting (*y*-axis), **(C)** clinical effective rate (*x*-axis), nausea and vomiting (*y*-axis), and leukopenia (*z*-axis), **(D)** performance status (*x*-axis), oral mucositis (*y*-axis), and leukopenia (*z*-axis), **(E)** leukopenia (*x*-axis), nausea and vomiting (*y*-axis), and thrombopenia (*z*-axis), **(F)** leukopenia (*x*-axis), oral mucositis (*y*-axis), and nausea and vomiting (*z*-axis). Note: (AD, Aidi injection; CCRT, concurrent chemoradiotherapy; CKS, Compound Kushen injection; EL, Elemene injection; KA, Kangai injection; KLT, Kanglaite injection; SM1, Shengmai injection; SQFZ, Shenqifuzheng injection; SM2, Shenmai injection; and XAP, Xiaoaiping injection).

The effects of the interventions on two different outcomes were comprehensively compared by cluster analysis. Eight interventions reported both the clinical effective rate and leukopenia. Compared with other interventions, Kangai injection + CCRT and Elemene injection + CCRT were similarly superior, and CCRT only produced the worst result. Furthermore, regarding the ARs of chemotherapy and radiotherapy, of eight interventions, Xiaoaiping injection + CCRT and Kangai injection + CCRT showed the most favorable benefits in terms of leukopenia and nausea and vomiting, while CCRT only yielded the worst result. Different colored dots indicate different types of interventions in Figure 5.

When cluster analysis was conducted on seven interventions that reported the clinical effective rate, leukopenia, and nausea and vomiting, Kangai injection + CCRT and Compound Kushen injection + CCRT had advantages in the ranking, while CCRT only had the worst ranking result. Similarly, in terms of the performance status, alleviation of leukopenia and grade ≥3 oral mucositis, Kangai injection + CCRT had the highest probability among the five interventions. Moreover, in the comprehensive ranking of leukopenia, thrombopenia, nausea and vomiting, and grade ≥3 oral mucositis among seven interventions, Kangai injection + CCRT had the highest probability, while CCRT only yielded the worst result. Different colored dots indicate different types of interventions in Figure 5.

### Publication Bias

**FIGURE 6:**
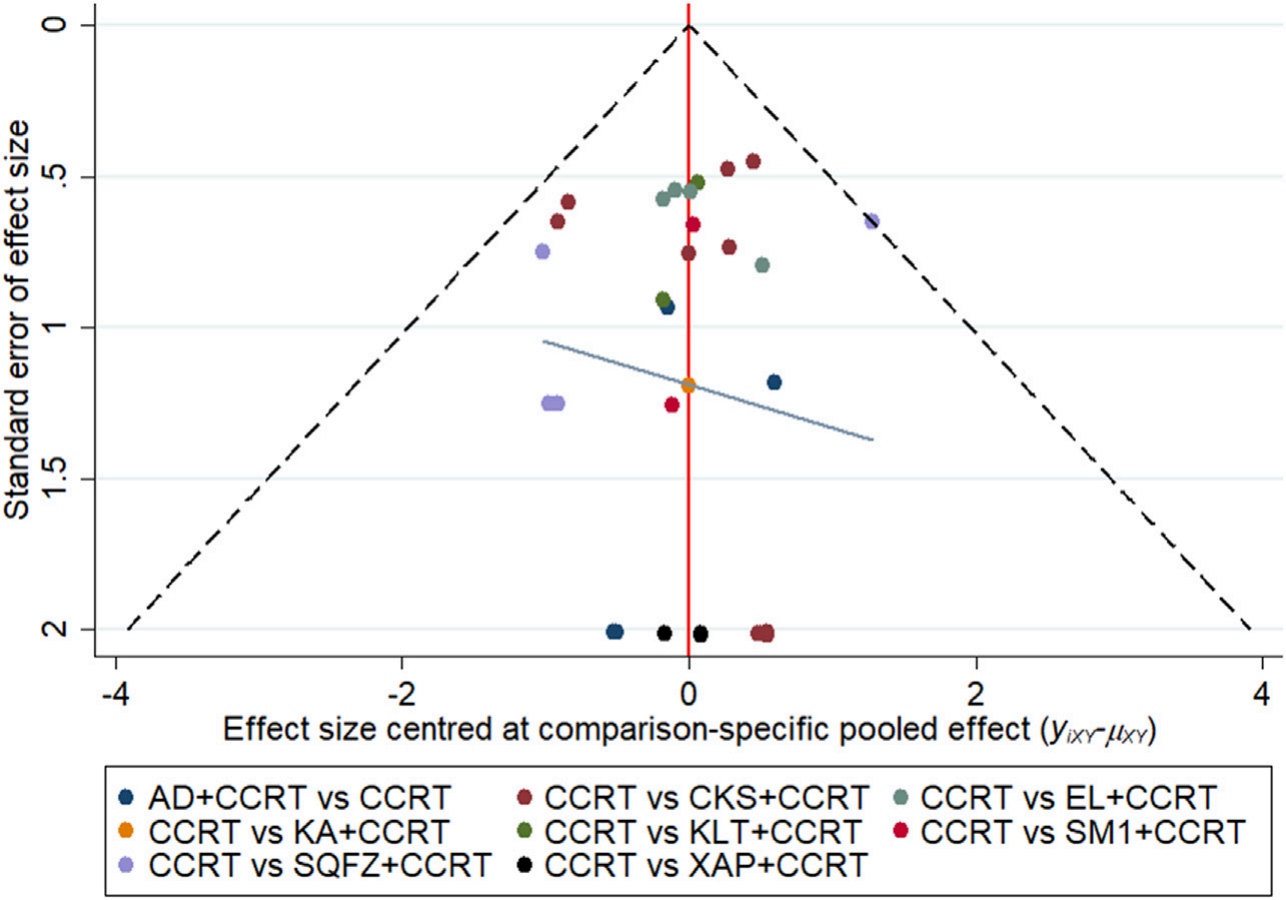
Funnel plots of clinical effective rate. Note: (AD, Aidi injection; CCRT, concurrent chemoradiotherapy; CKS, Compound Kushen injection; EL, Elemene injection; KA, Kangai injection; KLT, Kanglaite injection; SM1, Shengmai injection; SQFZ, Shenqifuzheng injection; SM2, Shenmai injection; and XAP, Xiaoaiping injection).

Comparison-adjusted funnel plots for the clinical effective rate were used to test publication bias. As depicted in Figure 6, certain angles between the correction auxiliary line and the centerline indicated that this study has potential publication bias.

## Discussion

To compare the efficacy outcomes of different CHIs combined with CCRT, this study used the NMA method to analyze evidence-based data from RCTs. Based on the results of the NMA, Elemene injection combined with CCRT was the best choice for improving the short-term clinical efficacy of the patients. Moreover, regarding improving the performance status, Shenqifuzheng injection combined with CCRT significantly ranked higher than the other injections. In terms of nausea and vomiting, Xiaoaiping injection was the best. In addition, Kangai injection combined with CCRT had the best effect on reducing leukopenia and thrombopenia, and Shengmai injection was likely to be the best choice for reducing grade ≥3 radiation-induced oral mucositis. However, since only two RCTs of Kangai injection and one RCT of SM2 injection were included in this study for analysis, its statistical efficiency was low. Therefore, the professional analysis of statistical results should be comprehensively considered. Moreover, Elemene injection showed a potential advantage based on the results of two outcomes, but more outcomes of related RCTs need to be observed and reported.

CCRT is the most commonly used method for advanced patients (Chen et al., 2019) because NPC is highly sensitive to radiotherapy (Blanchard et al., 2015). In this study, Compound Kushen injection and Elemene injection combined with CCRT showed a significant difference in improving the clinical effective rate compared with CCRT. Elemene injection is a preparation extracted from *Curcuma wenyujin* Y.H. Chen et C. Ling (Zingiberaceae; Curcumae radix), and β-elemene is the main ingredient that inhibits the growth of NPC cells (Wu et al., 2017). Compound Kushen injection is mainly prepared by extracting Sophora flavescens Aiton (Fabaceae; Sophorae flavescentis radix) and *Heterosmilax yunnanensis* Gagnep (Liliaceae), which can inhibit the growth of tumor cells (Wang et al., 2015), and its main components are matrine and oxymatrine. Furthermore, matrine and oxymatrine possibly inhibit NPC cell migration and invasion by suppressing the NF-κB pathway (Sun and Xu, 2015; Ni and Yi, 2017). As a preparation of traditional Chinese medicine, CHIs are often used as adjuvant treatments combined with chemoradiotherapy for NPC, which can reduce the incidence of ARs (Zhang et al., 2017). Kangai injection is mainly prepared from the extract from *Panax ginseng* C.A. Mey (Araliaceae; Ginseng radix et rhizome), *Astragalus mongholicus* Bunge (Fabaceae; Astragali radix), and matrine. The major ingredients of Shenqifuzheng injection are the extractives of *Codonopsis pilosula* (Franch.) Nannf (Campanulaceae; Codonopsis radix) and Astragali radix*.* Ginsenoside Rg3, an active pharmaceutical component extracted from ginseng, could inhibit the migration and invasion of NPC cells (Wang et al., 2019). Astragalus polysaccharide, an extract of Astragali radix, possibly inhibits NPC cell proliferation and induces apoptosis by modulating the expression of the Bax/Bcl-2 ratio and caspases to enhance the effect of cisplatin (Zhou et al., 2017). According to traditional Chinese medicine theory, tonic Chinese herbal medicine is commonly used to reduce the ARs of chemoradiotherapy. In addition, the effect of Kangai and Shenqifuzheng injection are both “*yi qi fu zheng*,” which means supplementing qi and strengthening the body, and the effect of Shenmai injection is *“yang yin sheng jin”,* which means nourishing *yin* to produce body fluid, corresponding to the statistical results of ARs (Zhang and Huang, 2019).

To our knowledge, this is the first NMA comparing the efficacy and safety of a variety of CHIs plus CCRT for NPC. A systematic review with an NMA reported different CHIs combined with radiotherapy (Yang et al., 2016). In that study, the best choice to improve the clinical effective rate was Kanglaite injection combined with radiotherapy, and Kangai injection combined with radiotherapy and Shenqifuzheng injection were the best choices in terms of oral mucositis. Excluding the different CHIs of the two studies, the remaining CHIs had a similar ranking for these two outcomes.

Notably, the limitations of the current NMA cannot be avoided. First, limited by the application scope of CHIs, all studies were performed in China, and all patients were Chinese. Thus, the results may not be generalizable. Second, 16 (43.24%) studies adequately reported the generation methods of random sequences, while none of included studies mentioned detailed information on allocation concealment and blinding methods, which may affect the reliability of the overall research. However, bias is unlikely to have a significant impact on the objective outcomes such as leukopenia and thrombopenia. Further, the survival rate is a critical indicator to judge the efficacy of treatment for cancer; however, only four (10.8%) studies reported the 3-years survival rate, and this study did not evaluate such long-term endpoint outcome indicators due to insufficient information to perform the NMA. Finally, due to the diversity of radiation and chemotherapy and the different doses and courses of CHIs, there was clinical heterogeneity. Therefore, we recommend that RCTs be registered in advance to ensure the transparency of the trial process and improve methodological quality. Despite the above limitations, the NMA provided a complete evaluation of the clinical efficacy of CHIs plus CCRT in multiple aspects.

## Conclusion

In summary, existing evidence shows that Elemene injection combined with CCRT has the best clinical effective rate and that Kangai injection might have a better impact on reducing adverse reactions when combined with CCRT in patients with NPC. In addition, due to the limitations of this NMA, more multicenter, high-quality, and head-to-head RCTs are needed to properly support our findings.

## Abbreviations

95% CI, 95% credible intervals; ARs, adverse reactions; CCRT, concurrent chemotherapy and radiotherapy; CHIs, Chinese herbal injections; KPS, Karnofsky performance status; MeSH, medical subject heading; NMA, network meta-analysis; NPC, nasopharyngeal carcinoma; OR, Odds ratio; PRISMA, the preferred reporting items for systematic reviews and meta-analyses; RCTs, randomized controlled trials; SUCRA, the surface under the cumulative ranking.
