# Supplementary material for: Investigation on the Efficiency of Chinese Herbal Injections combined with Concurrent Chemoradiotherapy for Treating Nasopharyngeal Carcinoma based on Multidimensional Bayesian Network Meta-analysis
Source: Front Pharmacol. 2021 Jun 11;12:656724. doi: 10.3389/fphar.2021.656724 (PMC8226160; doi:10.3389/fphar.2021.656724)
Supplement: Supplementary file 1 [file DataSheet1.docx]

Supplementary Materials

# File 1: PRISMA checklist for network meta-analysis.

**PRISMA checklist for the network meta-analysis**

| **Section/topic** | **#** | **Checklist item** | **Reported on page #** |
| --- | --- | --- | --- |
| **TITLE** | | |  |
| Title | 1 | Identify the report as a systematic review incorporating a network meta-analysis (or related form of  meta-analysis). | 1 |
| **ABSTRACT** | | |  |
| Structured summary | 2 | Provide a structured summary including, as applicable:  Background: main objectives  Methods: data sources; study eligibility criteria, participants, and interventions; study appraisal; and synthesis methods, such as network meta-analysis.  Results: number of studies and participants identified; summary estimates with corresponding confidence/credible intervals; treatment rankings may also be discussed. Authors may choose to summarize pairwise comparisons against a chosen treatment included in their analyses for brevity.  Discussion/Conclusions: limitations; conclusions and implications of findings.  Other: primary source of funding; systematic review registration number with registry name. | 2 |
| **INTRODUCTION** | | |  |
| Rationale | 3 | Describe the rationale for the review in the context of what is already known, including mention of why a network meta-analysis has been conducted | 2 |
| Objectives | 4 | Provide an explicit statement of questions being addressed with reference to participants, interventions, comparisons, outcomes, and study design (PICOS). | 2-3 |
| **METHODS** | | |  |
| Protocol and registration | 5 | Indicate if a review protocol exists and if and where it can be accessed (e.g., Web address), and, if available, provide registration information including registration number. | / |
| Eligibility criteria | 6 | Specify study characteristics (e.g., PICOS, length of follow-up) and report characteristics (e.g., years considered, language, publication status) used as criteria for eligibility, giving rationale. Clearly describe eligible treatments included in the treatment network, and note whether any have been clustered or merged into the same node (with justification). | 3-4 |
| Information sources | 7 | Describe all information sources (e.g., databases with dates of coverage, contact with study authors to identify additional studies) in the search and date last searched. | 4 |
| Search | 8 | Present full electronic search strategy for at least one database, including any limits used, such that it could be repeated. | 4 |
| Study selection | 9 | State the process for selecting studies (i.e., screening, eligibility, included in systematic review, and, if applicable, included in the meta-analysis). | 4 |
| Data collection process | 10 | Describe method of data extraction from reports (e.g., piloted forms, independently, in duplicate) and any processes for obtaining and confirming data from investigators. | 4 |
| Data items | 11 | List and define all variables for which data were sought (e.g., PICOS, funding sources) and any assumptions and simplifications made. | 4 |
| Geometry of the network | S1 | Describe methods used to explore the geometry of the treatment network under study and potential biases related to it. This should include how the evidence base has been graphically summarized for presentation, and what characteristics were compiled and used to describe the evidence base to readers | 4 |
| Risk of bias within individual studies | 12 | Describe methods used for assessing risk of bias of individual studies (including specification of whether this was done at the study or outcome level), and how this information is to be used in any data synthesis. | 4 |
| Summary measures | 13 | State the principal summary measures (e.g., risk ratio, difference in means). Also describe the use of additional summary measures assessed, such as treatment rankings and surface under the cumulative ranking curve (SUCRA) values, as well as modified approaches used to present summary findings from meta-analyses. | 4-5 |
| Planned methods of analysis | 14 | Describe the methods of handling data and combining results of studies for each network meta-analysis. This should include, but not be limited to: Handling of multigroup trials; Selection of variance structure; Selection of prior distributions in Bayesian analyses; and Assessment of model fit. | 4-5 |
| Assessment of inconsistency | S2 | Describe the statistical methods used to evaluate the agreement of direct and indirect evidence in the treatment network(s) studied. Describe efforts taken to address its presence when found. | / |
| Risk of bias across studies | 15 | Specify any assessment of risk of bias that may affect the cumulative evidence (e.g., publication bias, selective reporting within studies) | 5 |
| Additional analyses | 16 | Describe methods of additional analyses if done, indicating which were prespecified. This may include, but not be limited to, the following: Sensitivity or subgroup analyses; Meta-regression analyses; Alternative formulations of the treatment network; and Use of alternative prior distributions for Bayesian analyses (if applicable). | 5 |
| **RESULTS** | | |  |
| Study selection | 17 | Give numbers of studies screened, assessed for eligibility, and included in the review, with reasons for exclusions at each stage, ideally with a flow diagram. | 5-6 |
| Presentation of network structure | S3 | Provide a network graph of the included studies to enable visualization of the geometry of the treatment network. | 6 |
| Summary of network geometry | S4 | Provide a brief overview of characteristics of the treatment network. This may include commentary on the abundance of trials and randomized patients for the different interventions and pairwise comparisons in the network, gaps of evidence in the treatment network, and potential biases reflected by the network structure. | 6 |
| Study characteristics | 18 | For each study, present characteristics for which data were extracted (e.g., study size, PICOS, follow-up period) and provide the citations. | 6 |
| Risk of bias within studies | 19 | Present data on risk of bias of each study and, if available, any outcome level assessment. | 6 |
| Results of individual studies | 20 | For all outcomes considered (benefits or harms), present, for each study: 1) simple summary data for each intervention group, and 2) effect estimates and confidence intervals. Modified approaches may be needed to deal with information from larger networks. | 6-9 |
| Synthesis of results | 21 | Present results of each meta-analysis done, including confidence/credible intervals. In larger networks, authors may focus on comparisons versus a particular comparator (e.g., placebo or standard care), with full findings presented in an appendix. League tables and forest plots may be considered to summarize pairwise comparisons. If additional summary measures were explored (such as treatment rankings), these should also be presented. | 6-9 |
| Exploration for inconsistency | S5 | Describe results from investigations of inconsistency. This may include such information as measures of model fit to compare consistency and inconsistency models, P values from statistical tests, or summary of inconsistency estimates from different parts of the treatment network. | / |
| Risk of bias across studies | 22 | Present results of any assessment of risk of bias across studies for the evidence base being studied. | 9 |
| Results of additional analyses | 23 | Give results of additional analyses, if done (e.g., sensitivity or subgroup analyses, meta-regression  analyses, alternative network geometries studied, alternative choice of prior distributions for  Bayesian analyses, and so forth). | 8-9 |
| **DISCUSSION** | | |  |
| Summary of evidence | 24 | Summarize the main findings, including the strength of evidence for each main outcome; consider their relevance to key groups (e.g., health care providers, researchers, and policymakers). | 9 |
| Limitations | 25 | Discuss limitations at study and outcome level (e.g., risk of bias), and at review level (e.g., incomplete retrieval of identified research, reporting bias). Comment on the validity of the assumptions, such as transitivity and consistency. Comment on any concerns regarding network geometry (e.g., avoidance of certain comparisons). | 10 |
| Conclusions | 26 | Provide a general interpretation of the results in the context of other evidence, and implications for future research. | 10 |
| **FUNDING** | | |  |
| Funding | 27 | Describe sources of funding for the systematic review and other support (e.g., supply of data); role of funders for the systematic review. This should also include information regarding whether funding has been received from manufacturers of treatments in the network and/or whether some of the authors are content experts with professional conflicts of interest that could affect use of treatments in the network. | 10-11 |

# File 2: Search strategy for network meta-analysis

## Search strategy of Chinese herbal injections.

| 序号 | 注射剂名称 | 注射液英文检索词 | 中文检索词 |
| --- | --- | --- | --- |
| 1 | 参麦注射液 | Shenmai | 参麦注射液OR参麦注射剂OR注射用参麦 |
| 2 | 得力生注射液 | Delisheng | 得力生注射液OR得力生注射剂OR得力生 |
| 3 | 黄芪注射液 | Astragalus injection OR Huangqi injection | 黄芪注射液OR黄芪注射剂OR注射用黄芪冻干粉 |
| 4 | 生脉注射液 | Shengmai | 生脉注射液OR生脉注射剂OR注射用生脉 |
| 5 | 蟾酥注射液 | Chansu OR Toad venom | 佳素OR史君轻OR蟾毒康OR蟾酥注射液OR蟾酥注射剂 |
| 6 | 香菇多糖注射液 | Xiangguduotang OR Lentinan | 力提能OR天地欣OR香菇多糖注射液OR香菇多糖注射剂OR注射用香菇多糖 |
| 7 | 华蟾素注射液 | Huachansu OR Cinobufacini | 华蟾素注射液OR华蟾素注射剂OR华蟾素 |
| 8 | 参附注射液 | Shenfu | 参附注射液OR参附注射剂OR注射用参附 |
| 9 | 鸦胆子油乳注射液 | Yadanziyouru OR Javanica oil emulsion | 安体康注射液OR鸦胆子油乳注射液OR鸦胆子油乳注射剂OR鸦胆子油乳 |
| 10 | 消癌平注射液 | Xiaoaiping OR Marsdenia Tenacissima | 通关藤提取物OR通关藤注射液OR消癌平注射液OR消癌平注射剂OR消癌平 |
| 11 | 康艾注射液 | Kangai | 康艾液OR康艾注射液OR康艾注射剂 |
| 12 | 榄香烯乳注射液 | Lanxiangxi OR Elemene injection | 榄香烯注射液OR榄香注射液OR β-榄香烯注射液OR榄香烯脂质体注射液OR榄香烯乳注射液 |
| 13 | 艾迪注射液 | Aidi | 艾迪注射液OR艾迪注射剂OR爱迪注射液OR爱迪注射剂OR注射用艾迪OR艾迪液 |
| 14 | 参芪扶正注射液 | Shenqifuzheng | 参芪扶正注射液OR参芪扶正注射剂OR参芪扶正 |
| 15 | 康莱特注射液 | Kanglaite | ZCE-3静脉乳OR薏苡仁提取液OR注射薏苡仁油OR薏苡仁酯OR康莱特注射液OR康莱特注射剂 |
| 16 | 复方苦参注射液 | Compound matrine OR Compound Kushen OR Fufangkushen OR yanshu | 岩舒注射液 OR岩舒OR复方苦参注射液OR复方苦参注射剂OR复方苦参 |

## Search strategy of Pubmed.

#1 "Nasopharyngeal Neoplasms"[Mesh]
#2 Nasopharyngeal Neoplasm[Title/Abstract]
#3 Neoplasm, Nasopharyngeal[Title/Abstract]
#4 Neoplasms, Nasopharyngeal[Title/Abstract]
#5 Nasopharynx Neoplasm[Title/Abstract]
#6 Nasopharynx Neoplasms[Title/Abstract]
#7 "Cancer of Nasopharynx"[Title/Abstract]
#8 Neoplasms, Nasopharynx[Title/Abstract]
#9 Neoplasm, Nasopharynx[Title/Abstract]
#10 Cancers, Nasopharyngeal[Title/Abstract]
#11 Cancer, Nasopharyngeal[Title/Abstract]
#12 Nasopharyngeal Cancer[Title/Abstract]
#13 Nasopharyngeal Cancers[Title/Abstract]
#14 Cancers, Nasopharynx[Title/Abstract]
#15 Cancer, Nasopharynx[Title/Abstract]
#16 Carcinoma, Nasopharyngeal[Title/Abstract]
#17 Carcinomas, Nasopharyngeal[Title/Abstract]
#18 Nasopharyngeal Carcinomas[Title/Abstract]
#19 Nasopharyngeal Carcinoma[Title/Abstract]
#20 Nasopharynx Cancer [Title/Abstract]
#21 Nasopharynx Cancers[Title/Abstract]
#22 "Cancer of the Nasopharynx"[Title/Abstract]
#23 #1 OR #2 OR #3 OR #4 OR #5 OR #6 OR #7 OR #8 OR #9 OR #10 OR #11 OR #12 OR #13 OR #14 OR #15 OR #16 OR #17 OR #18 OR #19 OR #20 OR #21 OR #22
#24 Shenmai[All Fields]
#25 Delisheng[All Fields]
#26 Astragalus injection[All Fields]
#27 Huangqi[All Fields]
#28 Shengmai[All Fields]
#29 Chansu[All Fields]
#30 Toad venom[All Fields]
#31 Xiangguduotang[All Fields]
#32 Lentinan[All Fields]
#33 Huachansu[All Fields]
#34 Cinobufacini[All Fields]
#35 Shenfu[All Fields]
#36 Yadanziyouru[All Fields]
#37 Javanica oil emulsion[All Fields]
#38 Xiaoaiping[All Fields]
#39 Marsdenia Tenacissima[All Fields]
#40 Kangai[All Fields]
#41 Lanxiangxi[All Fields]
#42 Elemene[All Fields]
#43 Aidi[All Fields]
#44 Shenqifuzheng[All Fields]
#45 Kanglaite[All Fields]
#46 Compound matrine[All Fields]
#47 Fufangkushen[All Fields]
#48 Compound Kushen[All Fields]
#49 Yanshu [All Fields]
#50 #24 OR #25 OR #26 OR #27 OR #28 OR #29 OR #30 OR #31 OR #32 OR #33 OR #34 OR #35 OR #36 OR #37 OR #38 OR #39 OR #40 OR #41 OR #42 OR #43 OR #44 OR #45 OR #46 OR #47 OR #48 OR #49
#51 #23 AND #50

## Search strategy of Embase.

#1 'nasopharynx cancer'/exp
#2 Nasopharyngeal Neoplasm:ab,ti
#3 Neoplasm, Nasopharyngeal:ab,ti
#4 Neoplasms, Nasopharyngeal:ab,ti
#5 Nasopharynx Neoplasm:ab,ti
#6 Nasopharynx Neoplasms:ab,ti
#7 "Cancer of Nasopharynx":ab,ti
#8 Neoplasms, Nasopharynx:ab,ti
#9 Neoplasm, Nasopharynx:ab,ti
#10 Cancers, Nasopharyngeal:ab,ti
#11 Cancer, Nasopharyngeal:ab,ti
#12 Nasopharyngeal Cancer:ab,ti
#13 Nasopharyngeal Cancers:ab,ti
#14 Cancers, Nasopharynx:ab,ti
#15 Cancer, Nasopharynx:ab,ti
#16 Carcinoma, Nasopharyngeal:ab,ti
#17 Carcinomas, Nasopharyngeal:ab,ti
#18 Nasopharyngeal Carcinomas:ab,ti
#19 Nasopharyngeal Carcinoma:ab,ti
#20 Nasopharyngeal Neoplasms
#21 Nasopharynx Cancers:ab,ti
#22 "Cancer of the Nasopharynx":ab,ti
#23 #1 OR #2 OR #3 OR #4 OR #5 OR #6 OR #7 OR #8 OR #9 OR #10 OR #11 OR #12 OR #13 OR #14 OR #15 OR #16 OR #17 OR #18 OR #19 OR #20 OR #21 OR #22
#24 Shenmai
#25 Delisheng
#26 Astragalus injection
#27 Huangqi injection
#28 Shengmai
#29 Chansu
#30 Toad venom
#31 Xiangguduotang
#32 Lentinan
#33 Huachansu
#34 Cinobufacini
#35 Shenfu
#36 Yadanziyouru
#37 Javanica oil emulsion
#38 Xiaoaiping
#39 Marsdenia Tenacissima
#40 Kangai
#41 Lanxiangxi
#42 Elemene
#43 Aidi
#44 Shenqifuzheng
#45 Kanglaite
#46 Compound matrine
#47 Fufangkushen
#48 Compound Kushen
#49 Yanshu
#50 #24 OR #25 OR #26 OR #27 OR #28 OR #29 OR #30 OR #31 OR #32 OR #33 OR #34 OR #35 OR #36 OR #37 OR #38 OR #39 OR #40 OR #41 OR #42 OR #43 OR #44 OR #45 OR #46 OR #47 OR #48 OR #49
#51 #23 AND #50

## Search strategy of Cochrane Library.

#1 MeSH descriptor: [Nasopharyngeal Neoplasms] explode all trees
#2 Nasopharyngeal:ti,ab,kw
#3 Nasopharynx:ti,ab,kw
#4 Neoplasm*:ti,ab,kw
#5 Cancer*:ti,ab,kw
#6 Carcinoma*:ti,ab,kw
#7 Tumor*: ti,ab,kw
#8 Tumour*: ti,ab,kw
#9 #1 OR #2 OR #3
#10 #4 OR #5 OR #6 OR #7 OR #8
#11 #9 AND #10
#12 Delisheng:ti,ab,kw
#13 Astragalus injection:ti,ab,kw
#14 Huangqi injection:ti,ab,kw
#15 Shengmai:ti,ab,kw
#16 Chansu:ti,ab,kw
#17 Toad venom:ti,ab,kw
#18 Xiangguduotang:ti,ab,kw
#19 Lentinan:ti,ab,kw
#20 Huachansu:ti,ab,kw
#21 Cinobufacini:ti,ab,kw
#22 Shenfu:ti,ab,kw
#23 Yadanziyouru:ti,ab,kw
#24 Javanica oil emulsion:ti,ab,kw
#25 Xiaoaiping:ti,ab,kw
#26 Marsdenia Tenacissima:ti,ab,kw
#27 Kangai:ti,ab,kw
#28 Lanxiangxi:ti,ab,kw
#29 Elemene:ti,ab,kw
#30 Aidi:ti,ab,kw
#31 Shenqifuzhen:ti,ab,kw g
#32 Kanglaite:ti,ab,kw
#33 Compound matrine:ti,ab,kw
#34 Fufangkushen:ti,ab,kw
#35 Compound Kushen:ti,ab,kw
#36 Yanshu:ti,ab,kw
#37 Shenmai:ti,ab,kw
#38 #12 OR #13 OR #14 OR #15 OR #16 OR #17 OR #18 OR #19 OR #20 OR #21 OR #22 OR #23 OR #24 OR #25 OR #26 OR #27 OR #28 OR #29 OR #30 OR #31 OR #32 OR #33 OR #34 OR #35 OR #36 OR #37
#39 #11 AND #38

# File 3: Detailed information on included CHIs

|  | **Name of injection** | **Source** | **Species /Raw materials** | **Botanical plant names** | **Function** | **Indication** | **Quality control reported? (Y/N)** |
| --- | --- | --- | --- | --- | --- | --- | --- |
| 1 | Aidi injection | Guizhou Yibai Pharmaceutical Co., Ltd. | Mylabris 1.5g (animal drug), Ginseng Radix Et Rhizoma 50g, Astmgali Radix 100g, Acanthopanacis Senticosi Radix Et Rhizoma Seu Caulis 150g, Astmgali Radix 20g | *Panax ginseng* C.A.Mey*.;* *Astragalus mongholicus* Bunge; *Eleutherococcus senticosus* (Rupr. & Maxim.) Maxim | Clearing heat and detoxifying, dissipating blood stasis and removing knots | Primary liver cancer, lung cancer, rectal cancer, malignant lymphoma, gynecological malignant tumors, etc. | Y-National Pharmaceutical Standard Z52020236; Standard number: WS_3_-B-3809-99-2002 |
| 2 | Compound kushen injection | Shanxi Zhendong Pharmaceutical Co., Ltd. | Radix Sophorae Flavescentis 1400g, Heterosmilacis Rhizoma 600g | *Sophora flavescens* Aiton; *Heterosmliax yunnanensis* Gagnep. | Clearing heat and dampness, cooling blood and detoxifying, dispersing knots and relieving pain | Cancer pain and bleeding | Y-National Pharmaceutical Standard Z14021231, Standard number: WS_3_-B-2752-97 |
| 3 | Elemene injection | Dalian Holly Kingkong Pharmaceutical Co., Ltd. | β-elemene, γ-elemene, δ-elemene | / | / | Glioma and brain metastases, adjuvant therapy of cancerous pleural effusions and ascites | Y-National Pharmaceutical Standard H10960114, Standard number:WS_1_-(X-095)-2000Z |
| 4 | Kangai injection | Changbaishan Pharmaceutical Co., Ltd. | Astragali Radix 300g, Ginseng Radix Et Rhizoma 100g, Matrine 10g (Chemical medicine) | *Astragalus mongholicus* Bunge*;* *Panax ginseng* C.A.Mey. | Invigorating Qi, strengthening immune function of the body | Primary liver cancer, lung cancer, rectal cancer, malignant lymphoma and gynecological malignant tumors; leukopenia and hypoxia caused by various causes; chronic hepatitis B | Y-National Pharmaceutical Standard Z20026868, Standard number: Ws-11222 (ZD-1222) -2002 |
| 5 | Kanglaite injection | Zhejiang Kanglaite Pharmaceutical Co., Ltd. | Coicis Semen oil | *Coix lacryma-jobi* L. | Reinforcing Qi, nourishing Yin, eliminating symptoms and resolving knots | Primary non-small cell lung cancer and primary liver cancer with deficiency of both qi and Yin and dampness due to spleen deficiency should not be operated on; combined with radiotherapy and chemotherapy, it has certain enhancement operation; it has certain anti-cachexia and analgesic effect for patients with advanced cancer. | Y-National Pharmaceutical Standard Z10970091, National Food and Drug Administration National Drug Standard WS3-301 (Z-038) -2006 (Z) -2013 |
| 6 | Shenmai injection | Ya'an Sanjiu Pharmaceutical Co., Ltd.;  Zhengda Qingchunbao Pharmaceutical Co., Ltd.;  Hebei Shenwei Pharmaceutical Co., Ltd.;  Sichuan Chuanda West China Pharmaceutical Co., Ltd.;  Yunnan Gejiu Biopharmaceutical Co., Ltd.;  Sichuan Shenghe Pharmaceutical Co., Ltd.;  Dali Pharmaceutical Co., Ltd. | Red Ginseng, Radix Ophiopogonis | *Panax ginseng* C.A.Mey.*;* *Ophiopogon japonicus* (Thunb.) Ker Gawl | Reinforcing Qi and Restoring Yang、replenishing Yin to promote production of body fluids and generating meridian | Shock of deficiency of both qi and yin, coronary heart virus myocarditis, chronic cor pulmonale and granulocytopenia can improve the immune function of cancer patients, enhance their immune function when combined with chemotherapeutic drugs, and reduce the toxic and side effects caused by chemotherapeutic drugs | Y-National Pharmaceutical Standard Z51021845, Z33020019, Z13020889, Z51021353, Z53021720, Z51021263, Z20093649; National Food and Drug Administration National Drug Standard WS3-B-3428-98-2010Z |
| 7 | Shengmai injection | Changshu Leiyunshang Pharmaceutical Co., Ltd.;  Shanghai Hutchison Pharmaceutical Co., Ltd.;  Ya'an Sanjiu Pharmaceutical Co., Ltd.;  Jiangsu suzhong pharmaceutical group co. LTD.;  Sichuan Chuanda West China Pharmaceutical Co., Ltd.;  Wuliangye Group Yibin Pharmaceutical Co., Ltd.;  Shanxi Taihang Pharmaceutical Co., Ltd.;  Jilin Jian Yisheng Pharmaceutical Co., Ltd. | Red Ginseng 100g, Radix Ophiopogonis 312g, Schisandrae Chinensis Fructus 156g | *Panax ginseng* C.A.Mey.*;* *Ophiopogon japonicus* (Thunb.) Ker Gawl.*;* *Schisandra chinensis* (Turcz.) Baill | Reinforcing Qi, nourishing Yin, restoring meridian and securing the prolapse | Palpitation, shortness of breath, cold limbs, sweating, desperate pulse and myocardial infarction, cardiogenic shock, septic shock according to the above syndromes | Y-National Pharmaceutical Standard Z20044155, Z31020219, Z51021882, Z20053993, Z51021356, Z51022475, Z14020812, Z22025251; Executive Standard WS3-B-2865-98 |
| 8 | Shenqifuzheng injection | Livzon Group Limin Pharmaceutical Factory | Codonopsis Radix, Astmgali Radix | *Codonopsis pilosula* (Franch.) Nannf.; *Astragalus mongholicus* Bunge | Reinforcing Qi and reinforcing the healthy Qi | Spleen-lung Qi deficiency causes fatigue, lack of Qi and laziness, and dizziness due to spontaneous sweating. Auxiliary treatment for lung cancer and gastric cancer with the above symptoms | Y-National Pharmaceutical Standard Z19990065, Standard Number WS3-387(Z-50)-2003(Z)-2011 |
| 9 | Xiaoaiping injection | Tonghua Jinma Pharmaceutical Group Co., Ltd.;  Nanjing Shenghe Pharmaceutical Co., Ltd. | Marsdeniae Tenacissimae Caulis 200ml | *Marsdenia tenacissima* (Roxb.) Moon | Clearing heat and detoxifying, resolving phlegm and softening hard mass | Esophageal cancer, gastric cancer, lung cancer and liver cancer can be treated with adjuvant therapy of radiotherapy and chemotherapy | Y-National Pharmaceutical Standard Z20025869, Z20025868; Standard Number WS-10630-(ZD-0630)-2002 |
